# The Role of Tumor Necrosis Factor in Manipulating the Immunological Response of Tumor Microenvironment

**DOI:** 10.3389/fimmu.2021.656908

**Published:** 2021-04-27

**Authors:** Dipranjan Laha, Robert Grant, Prachi Mishra, Naris Nilubol

**Affiliations:** Surgical Oncology Program, Center for Cancer Research, National Cancer Institute, NIH, Bethesda, MD, United States

**Keywords:** tumor microenvironment, tumor necrosis factor-α, transforming growth factor beta, extracellular matrix, interstitial fluid pressure

## Abstract

The tumor microenvironment (TME) is an intricate system within solid neoplasms. In this review, we aim to provide an updated insight into the TME with a focus on the effects of tumor necrosis factor-α (TNF-α) on its various components and the use of TNF-α to improve the efficiency of drug delivery. The TME comprises the supporting structure of the tumor, such as its extracellular matrix and vasculature. In addition to cancer cells and cancer stem cells, the TME contains various other cell types, including pericytes, tumor-associated fibroblasts, smooth muscle cells, and immune cells. These cells produce signaling molecules such as growth factors, cytokines, hormones, and extracellular matrix proteins. This review summarizes the intricate balance between pro-oncogenic and tumor-suppressive functions that various non-tumor cells within the TME exert. We focused on the interaction between tumor cells and immune cells in the TME that plays an essential role in regulating the immune response, tumorigenesis, invasion, and metastasis. The multifunctional cytokine, TNF-α, plays essential roles in diverse cellular events within the TME. The uses of TNF-α in cancer treatment and to facilitate cancer drug delivery are discussed. The effects of TNF-α on tumor neovasculature and tumor interstitial fluid pressure that improve treatment efficacy are summarized.

## Introduction

The tumor microenvironment (TME) is a complex biological ecosystem of solid tumors encompassing all the cells and structures found in healthy organ tissue (1). These include, but are not limited to, blood vessels, immune infiltrates, fibroblasts, and the extracellular matrix. Tumor cells, immune cells, fibroblasts, myofibroblasts, and microvascular structures such as vascular endothelial cells and pericytes found within the TME play various critical roles in regulating tumor initiation and progression (1–3). These cell types can control tumor growth through their normal regulatory functions. However, the dysregulation of these cells can promote tumor growth and metastasis. Recent studies demonstrate that the relationships between cancer cells and their surrounding microenvironments affect cancer cell survival, growth, proliferation, epithelial-mesenchymal transition (EMT), and metastasis (4). Thus, the modulation of the TME as a target for clinical applications is an area of interest in cancer treatments. Communication between neoplastic cells and the TME is conducted mainly through standard mechanisms observed in communication between healthy cells in normal organ tissue: through intercellular junctions and receptor-signal pathways encased in a three-dimensional extracellular matrix (ECM). Glycoproteins, proteoglycans, cytokines, and growth factors provide structural support and information exchange (5). In both normal tissue and solid cancers, TNF-α has diverse regulatory roles in the TME depending on the type of cells.

TNF-α is, to a large degree, produced by macrophages but also by other immune cell types, including lymphoid cells, mast cells, and by non-immune cells such as endothelial cells, fibroblasts, and smooth muscle cells (6–9). The members of the TNF-α family exert their effects through two distinct receptors, TNFRSF1A (TNFR1) and TNFRSF1B (TNFR2). TNFR1 is ubiquitously expressed and found in all cell types. Among many diverse effects induced by TNF-α, the major role of TNFR1 is the initiation of apoptosis through its death domain, which is not found on TNFR2 (9). Seemingly contradictory to its apoptotic signaling function, activation of TNFR1 also can induce cell survival mechanisms (9). The determination of the final downstream activity of this receptor is based on the concentration of TNF-α in the microenvironment as well as the effects of other involved cytokines. TNFR2 is mostly found on immune cells, where its pathway activation by TNF-α assists in regulating the immune response and inflammation. TNFR2 activation on immune cells within the TME and cancer cells themselves can promote tumor growth and progression (10). Increased TNFR2 expression found on regulatory T cells within the TME can suppress the immune response and prevent activation of cytotoxic T cells, which decreases the ability of the immune response to suppress the tumor (10, 11). Myeloid-derived suppressor (MDSC) are a group of immature cells that can differentiate into several different immune cell types, but in their immature state, are potently immunosuppressive (10). TNF-α can suppress the MDSC differentiation and induce accumulation of MDSC, which enhances their immunosuppressive effects in the TME through TNFR2 signaling (10, 12, 13).

Through its roles in apoptosis, angiogenesis, and immune cell recruitment and regulation, as well as its function in assisting with the construction of the ECM, this review summarizes many roles of TNF-α and its relation to the various components of the TME. Many cell-signaling mechanisms are involved in these functions, and we attempt to explain the roles of these pathways in relation to this versatile cytokine. Through the understanding of these pathways, scientists and clinicians are finding ways to exploit them as therapeutic targets. For example, inhibition of endogenous TNF-α is a standard of care for chronic inflammatory diseases such as ulcerative colitis, Crohn’s disease, rheumatoid arthritis, and several other diseases. In addition, TNF-α was previously used with good efficacy in patients with limb soft tissue sarcomas (STS) and in-transit melanoma by targeting the hap-hazard neovascular growth within the TME of these lesions (14–16). In this review, it is evident that there are many potential applications to manipulate TNF-α pathways, specifically in its role in the TME for cancer therapy.

## TNF-α Pathway at Glance

Research dating back more than twenty years has shown that the TNF-α superfamily comprises at least 19 members that signal through 29 receptors (17). It is a pleiotropic cytokine, binds two receptors – TNFR1(receptor type 1: CD120a; p55/60) and TNRFR2 (TNF receptor type 2; CD120b; p75/80) – and is produced by many different types of cells (17). Unlike the TNFR2 expression which is limited to immune cells and a few other cell types, TNFR1 expression is present in various cell types (18–20). TNF-α binds to these receptors resulting in several diverse effects, cell proliferation, survival, and apoptosis (21–23). Abnormal production of TNF-α and TNF receptor signaling has been associated with the pathogenesis of several inflammatory diseases including rheumatoid arthritis, Crohn’s disease, atherosclerosis, psoriasis, and cancer (24). TNF-α has both tumor-promoting and tumor-suppressing roles in the TME. It is well reported that the tumor parenchyma and the TME continuously produce endogenous TNF-α, which induces tumor angiogenesis and promotes progression. The innate immune cells of the TME secrete various cytokines such as TNF-α and interleukin-6 (IL-6), which can promote cancer cell survival (25) and induce the expression of vascular endothelial cell adhesion molecules (CAM) that facilitate extravasation of leukocytes (26). TNF-α mediated matrix metalloproteinase (MMP) production in tumor cells or the TME also promotes tumor expansion (27, 28).

TNF-α plays an important role in tumor metastasis. It increases the expression of angiogenic factors such as basic fibroblast growth factor (bFGF), interleukin-8 (IL-8), and vascular endothelial growth factor (VEGF) in endothelial cells of the TME. TNF-α induced the expression of adhesion molecules such as intracellular adhesion molecule (ICAM)-1, E-selectin, and VCAM-1 in liver sinusoidal endothelial cells and induced tumor metastasis (29). So far, several FDA-approved TNF-α inhibitors, such as infliximab, etanercept, and adalimumab have been used to treat various human illnesses (30). We have summarized the multiple roles of TNF-α in different solid cancers based on preclinical studies (**Table 1**).

**Table 1:** The roles of TNF-α in different cancer types.

Cancer Type	Known TNF-α target pathways
Prostate cancer	Induce pro-survival signaling in androgen-dependent prostate cancer (31) Sensitize the cells to irradiation to induced apoptosis in LNCaP cells (32) Induce apoptosis in androgen-sensitive and insensitive LNCaP and PC-3, respectively (33)
Breast Cancer	Promotes the growth of breast cancer in MDA-MB 468 and SK-BR3 cells (34) Inhibits proliferation and tumorigenesis (35)
Lung Cancer	Induce apoptosis in H292 and H1975 cell lines (36) Induce cell necrosis in H460 cells (37)
Melanoma	Inhibits apoptosis in A375, WM266.4, and Colo829 (38)
Cervical Cancer	Induce apoptotic cell death in cervical cancer cells (39)
Ovarian Cancer	Induce apoptotic cell death (40)
Hepatocellular carcinoma	Induce EMT (41)

## TNF-α Mediated Signaling Pathways in the TME

Two different forms of TNF-α have been identified: (i) soluble TNF-α (sTNF-α) and (ii) transmembrane TNF-α (mTNF-α) (42). The mTNF-α is the precursor of sTNF-α. TNF-α converting enzyme (TACE) can cleave mTNF-α, releasing sTNF-α. Previous findings reported dual roles of TNF-α based on the exposure time and cytokine levels reached within the TME (33). Soluble TNF-α mostly binds with TNFR1 and controls the inflammatory immune response, whereas mTNF-α mostly binds with TNFR2 and controls cell proliferation, survival, and other biological activities. The interaction between mTNF-α and TNFR2 is dependent on responses to different signaling pathways. In colorectal cancer, TNFR2 modulates the expression of Ki67, influences fibroblast associated protein and α-smooth muscle actin, and increases cellular proliferation and migration (43). Anti-TNFR2 antibodies suppress tumor-associated regulatory T cells (Tregs) and inhibit ovarian cancer cell proliferation (44). Ligand binding to TNFR2 leads to the activation of NF-κB and several kinase pathways, including JNK, p38, MAPK, ERK, and PI3K (45). Apart from NF-κB and kinase pathways, other processes and signaling pathways, such as EMT and TGF-β receptor-mediated signaling, are also critically regulated through TNF-α signaling (described in detail below). We have summarized different oncogenic signaling pathways such as β-catenin, STAT3, PI3K/PTEN/AKT/mTOR, p53, which are directly or indirectly regulated by TNF-α in **Table 2**.

**Table 2:** The roles of TNF-α in different transcription factors, cytokines, and signaling pathways by cancer type.

Cell type	Known TNF-α target genes	Effects
Breast Cancer (MDA-MB-231, MCF7, and HCC1937)	TIPE2 (35)	Induced
Lung Cancer (H292, H1299, H1975 and H460)	NF-κB (36)	Inhibited
Melanoma (A375, WM266.4, and Colo829)	BRAF (38)	Inhibited
Ovarian Cancer (SKOV-3, MDAH-2774, OVCAR-3)	AKT (46)	Induced
Human Dermal β Fibroblast	TGF-β (47)	Inhibited
Prostate carcinoma cells (LNCap)	PI3K/AKT (48)	Inhibited
Hepatocellular carcinoma (HepG2, SK-Hep-1, L02, MHCC97-H, MHCC97-L, and Huh7)	PI3K (41)	Induced
Human colon carcinoma stem cells (HT29)	WNT (49)	Induced
Hepatocellular carcinoma (HepG2, SK-Hep-1, L02, MHCC97-H, MHCC97-L, and Huh7)	GSK 3-β/β-catenin (41)	Induced
Breast Cancer (MCF‐7 and MDA‐MB‐231)	Nur77 (50)	Induced
Breast cancer (MDA-MB-231 and MDA-MB-468)	CCL2 (51)	Induced
Gallbladder Cancer (GBC-SD and SGC-996)	ERK1/2/AP-1/VEGF-D (52)	Induced

## Role of TNF-α in TGF-β Receptor Mediated Signaling Pathways

The multifunctional cytokine transforming growth factor-beta (TGF-β) regulates cell growth, extracellular matrix protein synthesis, and immune cell functions (53). In normal and premalignant cells, TGF-β acts as a tumor suppressor through the induction of apoptosis. However, when cancer cells have mutations or lose tumor suppressor genes, cells become resistant TGF-β mediated growth arrest. The crucial role of the TGF-β signaling pathway in the TME has been demonstrated in several studies. Several molecules regulate the TGF-β pathway, among them, TNF-α is of significant importance. However, it is not clear whether TNF-α directly or indirectly interacts with the TGF-β pathway. Understanding the molecular mechanisms of the antagonistic activities of TNF-α against TGF-β is critical. Studies have demonstrated that TNF-α inhibits TGF-β and ECM production, such as type I collagen and elastin in cancer cells and fibroblasts (47, 54, 55). TGF-β inhibits cancer growth through the activation of tumor angiogenesis and regulates prominent compounds involved with cancer-associated fibroblasts (CAF) in TME (56). Fibroblasts have been shown to facilitate cancer progression by supporting tumor growth, extracellular matrix remodeling, angiogenesis and by mediating tumor-promoted inflammation (57). Recent research has clarified the relationship between TGF-β regulation mediated by TNF-α and CAF. In brief, TNF-α triggers the downregulation of TGF-β receptor II leading to desensitization of human dermal fibroblasts toward TGF-β. Additionally, TNF-α impaired the response of the cells to TGF-β by regulating the turnover of TRII (47). Normal fibroblasts acquire characteristics of CAFs when stimulated with TNF-α. The most widely used marker for CAFs is α-smooth muscle actin (α-SMA). TGF-β induces α-smooth muscle actin expression in fibroblasts (58).

## Role of TNF-α in NF-κB Pathways

The TME contains various types of cells, including tumor-associated macrophage (TAMs), dendritic cells, myeloid-derived suppressor cells, neutrophils, mast cells, natural killer T (NKT) cells, cancer-associated fibroblast (CAFs) and endothelial cells (59). The nuclear factor kappa-light chain enhancer of activated B cells (NF-κB) functions in these cell types and modulates inflammation, tumorigenesis, and metastasis. NF-κB activates several inflammatory genes in response to cytokines like TNF-α and IL-1, as well as to bacterial endotoxin and physical stress. Whiteside reported NF-κB activation in myeloid cells enhances inflammation in the TME (60). When cells are stimulated by an extracellular signal such as TNF-α, NF-κB is activated and enters the nucleus to bind to target genes and promotes cell death or increases cell survival in a context-dependent manner (61). In lung carcinoma cells, deoxynivalenol induces dependent proteolytic cleavage of TNFR1 through the activation of ERK and p38 MAPK, and subsequently inhibits the TNF-α-induced NF-κB signaling pathway (62). Tang et al. reported that in oral squamous cell carcinoma, TNF-α activates the NF-κB pathway, which promotes invasion and metastasis (63). In addition, NF-κB is also an important player in modulating the tumor-associated macrophages (TAM). The NF- κB pathway regulates anti and pro-inflammatory signaling pathways in the TME through tumor-associated macrophage (TAM) regulation.

## Role of TNF-α in the Receptor Tyrosine Kinase Pathway

Receptor tyrosine kinases (RTK) act as a mediator between the extracellular and intracellular compartments by transferring signals from the TME into the tumor cells (64). So far, 58 different RTKs have been discovered in humans and classified into 20 different subfamilies based on their structural features, and activation of these enzymes is well defined to regulates various cellular processes (64). Mutations in RTKs and their associated downstream signaling pathways have oncogenic roles in many solid cancers, hence the development of targeted therapy specifically for these RTKs (64). However, many cancer types often acquire treatment resistance to various RTK inhibitors such as VEGFR inhibitors (bevacizumab), EGFR inhibitors (gefitinib), and FGFR inhibitors (AZD4547) (65). The mechanism associated with RTK inhibitors to disrupt neoplastic cellular growth are (i) immunogenic modulation of the TME and (ii) immune subset conditioning (66). RTK inhibitors induce immunogenic modulation *via* tumor cell sensitivity to immune cells-mediated lysis through an alternation in tumor cell phenotype and cause immune subset conditioning by activating immune cells and suppressing the immune suppressor cells in the TME (66).

TNF-α regulates multiple RTK pathways, including mitogen-activated protein kinases (MAPK) (i) extracellular-signal-regulated kinases (ERKs); (ii) cJun NH_2_-terminal kinases (JNK); and (iii) p38 MAP kinases pathways. Downregulation of MAP2K isoforms MKK4 and MKK7 in mice model prevents TNF-α mediated JNK activation (67, 68).

Apart from MAPK pathways, TNF-α signaling also controls vascular endothelial growth factor receptor (VEGFR), an RTK, which influences angiogenesis in the TME (69). This happens through TNFR1 signaling inflammatory macrophages to upregulate expression of vascular endothelial growth factor receptor 3, which causes increased production of vascular endothelial growth factor-C, and in turn, induces angiogenesis (69). The result was validated *in vivo* in mice with loss of function of TNFR1 (Tnfr1(-/-)) which reduced lymphangiogenesis and lymphatic metastasis (69). Tumor-mediated TNF-α and VEGF production is also associated with integrin receptor alpha V and beta 3 and beta 5 (αvβ3/αvβ5) mediated neovascularization, which shows an active interaction between tumor cells and endothelial cells through TNF-α (70). Another study demonstrated that a weakness of Akt/NF-κB signaling from TNF-α-mediated cross-talk signaling *via* EGFR causes the collateral sensitivity to TNF-α in a gefitinib resistant cell line (71). For instance, the over-expressions of EGFR and platelet-derived growth factor receptor α/β have been explored in tumor growth and progression (72). Sasi et al. confirmed that blocking of TNFR2/p75 with short-hairpin RNA in a Lewis Lung Carcinoma cell line induced apoptosis and decreased expression of the angiogenic growth factors VEGF, HGF, and PLGF (73). The VEGF inhibitor bevacizumab regulates a process called vessel normalization during angiogenesis through the upregulation tumor-infiltrating lymphocytes such as CD4+ and CD8+T cells in the TME (73). Below we more thoroughly discuss how TNF-α regulates T cells in the TME.

## TNF-α and Epithelial-Mesenchymal Transition Regulatory Molecules

The epithelial to mesenchymal transition (EMT) is a process whereby epithelial cells lose epithelial features and acquire properties of mesenchymal cells. The EMT is classified into three main types depending upon the biological context. Type I EMT is observed during embryonic development, Type II occurs during wound healing and tissue regeneration, and Type III occurs during cancer progression. Previous findings demonstrated that TNF-α had been implicated as a major factor in EMT through cancer initiation and progression in the TME (6, 41, 74). Wang et al. showed that TNF-α induces EMT in human HCT116 cells and promotes colorectal cancer invasion and metastasis (75). The zinc finger protein *SNAI1* plays a crucial role in TNF-α induced EMT. TNF-α treatment increased the expression of *SNAI1* but not *SLUG, ZEB1*or *Twist.* Overexpression of *SNAI1*induced a switch from E-cadherin to N-cadherin expression in HCT116 cells, which is a characteristic of EMT. Recent findings from Li et al. showed that TNF-α mediated NF-κB activation upregulates EMT regulatory gene *TWIST1* expression in breast cancer cells (76). Mikesh et al. discovered the expression of molecular markers of mesenchymal phenotype in melanoma. Melanoma cells were treated with TNF-α in a 3-dimensional culture system, and the changes in the expression of E-cadherin, N-cadherin, vimentin, and fibronectin were assessed. Melanoma cells treated with TNF-α reduced the epithelial marker E-cadherin and induced mesenchymal markers N-cadherin, vimentin, fibronectin (77). The role of TNF-α in regulating the EMT in hepatocellular carcinoma cells (SMMC-7721) was studied by Chen et al. TNF-α is elevated in the supernatants of M2-tumor-associated macrophages (M2-TAMs), promoting the EMT of SMMC-7721 cells *in vitro (* 78).

## Extracellular Matrix and Tumor Microenvironment

The TME comprises various cell types embedded in an altered extracellular matrix (ECM). The ECM is a major structural component of the TME and is mainly produced by cancer-associated fibroblasts (CAF) (79). It is largely composed of fibrous proteins, glycoproteins, proteoglycans and polysaccharides (79). The ECM in solid tumors differs significantly from normal organs. During cancer progression, cancer cells cross the ECM and basement membrane. MMPs are a large family of calcium-dependent, zinc-containing endopeptidases which are proteolytic enzymes capable of degrading the macromolecules of the ECM (80). Cancer cells secrete many members of the MMP family that facilitate the cellular migration into the ECM and thereby causing local invasion and can lead to metastasis (80). This process is largely regulated by TNF-α. Dilshara et al. reported that mangiferin inhibits TNF-α induced MMP9 expression and cellular invasion by suppressing the NF-κB activity in human LNCaP prostate carcinoma cells (81). An isoquinoline derivative compound, berberine inhibits TNF-α induced MMP9 and cell invasion through the inhibition of AP-1 activity in MDA-MB-231human breast cancer cells (82).

Several studies demonstrated that the biologic phenotype of cancers not only depends upon the activities of cancer cells but also tumor-infiltrating immune cells in the TME. TNF-α is produced by macrophages and other immune cells, including dendritic cells, B cells, activated natural killer cells, and activated T cells (83, 84). We summarized the effects of TNF- α on various non-cancerous cells in the TME below (**Table 3**).

**Table 3:** The effect of TNF-α on various cell types in the TME.

Cell type	Effects
Macrophages	TNF-α secreted by M1 M2 phenotype inhibition
Neutrophils	Recruitment into the TME Priming and enhanced cytotoxicity
T cells	Enhances CD4^+^ memory Causes Treg accumulation and immunosuppression
Dendritic cells	Induce pro-tumorigenic CD4^+^ T cells

## Macrophages

The main function of a macrophage is to engulf and digest foreign substances, cellular debris, and other components of the TME. Once macrophages are recruited into the TME, they are polarized into M1 or M2 TAMs depending on the varying concentrations of cytokines in the TME. A high density of TAMs can be found in several cancers such as pancreatic (85), breast (86), ovarian (87), and esophageal (88) and is associated with adverse prognostic features (86, 89, 90). However, the story of TAMs is more sophisticated than simply the number of cells in the TME. There are two types of mature macrophages: (i) classically activated macrophages (M1) and (ii) alternatively activated macrophages (M2). M1 and M2 macrophages play an important role in immune regulation in the TME. TAMs are a significant producer of TNF-α within the TME and interestingly are also highly responsive to TNF-α. The M1 macrophage can be stimulated to secrete a high level of TNF-α, resulting in high concentrations of superoxide, free oxygen, and nitrogen radicals (91) which promotes cell death in TME. M2 TNF-α secretion has been shown to promote EMT and induce “stemness” in an *in vitro* hepatocellular carcinoma model (78). Porta et al. reported that the p50 subunit of NF-κB plays an important role in macrophage polarization both *in vitro* and *in vivo*. They concluded that the p50 homodimer inhibited the NF-κB signaling pathway and induced macrophages to display an M2 phenotype with reduced expression of TNF-α and inducible nitric oxide synthases (iNOS) (92). M2 macrophages produce anti-inflammatory cytokines such as IL-10, IL-13, and TGF-β that may facilitate tumor development in TME. Experimental therapies to date have focused on depletion of M2 macrophages in the TME, specifically in glioblastoma through the inhibition of the colony-stimulating factor-1 receptor, which skewed macrophage polarization in the TME away from M2 and toward M1 *in vivo* (93). Data from another group using multiple different tumor models *in vitro* and *in vivo* showed higher TNFR activation shifts the balance toward the M1 phenotype and partially inhibited gene expression, specifically characteristic of M2 TAMs (94).

## Neutrophils

Like macrophages, two types of neutrophil populations have been identified within the TME. First described by Fridlender et al. in mesothelioma *in vitro* and xenograft model, the tumor-associated neutrophils (TAN) are polarized into a subpopulation of anti-neoplastic (N1) or, through induction by TGF-β within the TME, pro-neoplastic (N2) neutrophils (95). While TNF-α has not been directly implicated in this polarization, its role in TGF-β pathway and the other pathways in which it modulates neutrophil activity described below highlights the importance of neutrophil-neoplasm interaction. In normal vasculature *in vivo*, TNF-α increases neutrophil recruitment and endothelial adhesion *via* cytoskeletal remodeling (96, 97). TNF-α also has a “priming” effect on neutrophils, causing them to be more responsive to stimuli (98). This priming causes a respiratory burst and the generation of intracellular reactive oxygen species (ROS) and reactive nitrogen species (RNS) for neutrophils to interact with pathogens and to modulate local inflammation. In *in vitro* model of pancreatic ductal adenocarcinoma, neutrophils promoted EMT and metastasis in co-culture (99). In these experiments, cancer cells caused neutrophils to secrete large amounts of TNF-α and TGF-β in a co-culture model, indicating that the cytokines were responsible for regulating the EMT and the metastasis (99). A recent study demonstrated higher levels of circulating TNF-α in patients with breast cancer. The neutrophils from these patients exhibit more cytotoxicity against breast cancer cell lines *ex-vivo* than that of the neutrophils from patients without breast cancer (100). This work also showed that neutrophils from patients with and without breast cancer exposed to TNF-α *ex-vivo* exhibited enhanced cytotoxicity, with even further cytotoxicity seen in patients with breast cancer (100). TNF-α and TANs interact in many ways: 1) TANs are recruited into tumors partially from the influence of TNF-α 2) T cells attract and prime TANs with TNF-α 3) N1 TANs can attract cytotoxic T cells by TNF-α secretion 4) TANs can activate dendritic cells with TNF-α and assist CD4+ T cells with anti-neoplastic memory (101). In summary, neutrophil secretion of and reaction to TNF-α causes many interactions in the TME may be exploited as potential targets in cancer therapy.

## T Cells

A heterogeneous population of tumor‐infiltrating lymphocytes, CD8^+^ cytotoxic T cells, CD4^+^ helper T cells, and regulatory T cells (Tregs) are present in the TME. As with both TAMs and TANs, these tumor-infiltrating lymphocytes either suppress or enhance tumor growth. Previous studies demonstrated that T cell responses are regulated by TNFR activation and mediated cross-talk between T cells and other cell types. The TNFR superfamily (TNFRSF) OX40, 4-1BB, CD27, and DR3 are associated with TNFR associated factors (TRAF). In detail, TRAFs can bind with the α subunit of NF-κB and I Kappa B Kinase Beta (IkB kinase-β) to assist in the activation of both canonical and non-canonical NF-κB signaling pathways. OX40, 4-1BB, and CD27 mediated activation of signaling pathways in CD4^+^ and CD8^+^ T cells increase the expression of the anti-apoptotic molecule BCL-2, which correlates with the promotion of T cell survival (102). Prior work has demonstrated that the treatment with recombinant human TNF-α in a B16F10 melanoma mouse model of lung metastasis increased tumor burden and metastatic foci and was associated with increased numbers of pulmonary regulatory CD4^+^/Foxp3^+^ T cells. TNF-α activates TNFR2 on Tregs which helps expand the immunosuppressive role of the immune cell population by inducing CD8^+^ and CD4^+^ T cells. But the accumulation of Tregs can be prevented through dysregulation of TNF-α or TNFR2 which creates less tolerogenic environment and prevents B16F10 tumor metastasis and growth (103). Hu et al. reported that TNFR2 progranulin induced the proliferation of suppressive mouse CD4^+^/Foxp3^+^ regulatory T cells (104). Therefore, the targeting of T cell-associated mechanisms has been considered a major strategy for cancer immunotherapy.

## Dendritic Cells

The antigen-presenting cells (APC), dendritic cells (DC) can take up, process, and present different types of antigens, including tumor antigens, to naïve T cells. This antigen presentation can induce the creation of tumor-specific cytotoxic T cells (105). DC can also downregulate the immune response or induce immune tolerance in the TME through exposure to different stimuli. For example, when exposed to thymic stromal lymphoprotein from tumor or thymus (105), or TNF-α (106), DC express OX40 ligand, which is a member of the TNF superfamily (TNFSF4). The T cell membrane OX40 binds to OX40L on DC and induces a phenotypic subtype of CD4^+^ T cells, promoting tumor growth (105). DC induced CD4^+^ T cells to polarize to this subtype which highly secreted IL-13, which promoted breast cancer progression *in vivo* (107). Conversely, a subset of inflammatory DC which secrete TNF-α and produce nitric oxide are known to be tumor suppressive (108, 109). *In vivo* adjunctive use of this inflammatory DC subtype was shown to potentiate the effect of adoptive cell transfer, allowing for a more potent treatment strategy for cancer immunotherapy (108).

## Role of TNF-α in Cancer Stem Cells

The cancer stem cells (CSC) is a subpopulation within a primary cancer that have the potential to differentiate into a more mature phenotype and exhibit properties of self-renewal and immortality. CSC were first described in acute myeloid leukemia (AML) from the identification of a subset of specific surface proteins, which were found to regenerate AML *in vivo* (110). Since this initial description, they have been described in many different solid cancers in addition to hematologic malignancies and are implicated in chemotherapy resistance and progression to metastasis (111). Accumulating evidence suggests that TNF-α has a role in CSC regulation. TNF-α increased the breast CSC population through NF-κB/HIF1α/Slug (112). Zhao et al. reported that TNF-α treatment in a colon cancer cell-line (NCM460) derived spheroids induced NF-κB and Wnt/β-catenin pathways which can accelerate malignant transformation in intestinal stem cells (113). Additionally, using an osteosarcoma cell line *in vitro*, Yao et al. showed TNF-α exposure upregulated a specific microRNA (miR-155) and found that miR-155 produced a stem cell-like phenotype which promoted cancer progression (114). Suggested by these studies, the role of TNF-α in CSC may contribute to tumorigenesis and progression. However, other TNF-α-related tumor-suppressive effects may counter the effects of TNF-α on CSC.

## TNF-α Mediated MicroRNA Regulation

MicroRNAs (miRNA) are short non-coding RNAs that regulate gene expression at the post-transcriptional level by binding to the 3’-untranslated region of their targeted mRNA resulting in the suppression of protein production. miRNAs are dysregulated in several cancer types. Thus, understanding the role of miRNAs in the TME is crucial (115). The remodeling of miRNAs in the TME has a role in tumor growth, metastasis, and resistance to treatment. Several miRNAs are released from neoplastic cells into the TME and regulate the functions of endothelial cells, immune cells, and fibroblasts. Previous studies from several groups reported that miR-145, miR-15a, miR-29a, miR-181A, miR-19a, miR-130a, miR-21, miR-765 are regulated by TNF-α in several cancers produced by both cancer cells and TME-related immune cells. Eleven miRNAs were shown to be differentially expressed between cancer-associated fibroblasts and healthy tissue, and 114 upregulated and 85 downregulated miRNAs have been identified in gastric cancer mesenchymal stem cells (GC-MSCs) (116). Zeng et al. showed that the tumor suppressor miR-145 is downregulated in triple-negative breast cancer cell lines MDA-MB-231, and when treated with TNF-α, this miRNA is overexpressed and induces apoptosis (117). Co-immunoprecipitation data revealed that miR-145 facilitates the formation of RIP1-FADD -caspase 8-mediated apoptotic complex with TNF-α treatment (118). Huang et al. showed that miR-19a is associated with lymph node metastasis and mediates TNF-α induced EMT in colorectal cancer (119). Furthermore, miR-19a is upregulated by TNF-α and miR-19a is required for TNF-α induced EMT and metastases in CRC cells (120). TNF-α also has a role in nuclear translocation of NF-κB followed by induction miR-130 and expression and downregulation of TNF-α (121). Higher levels of TNF-α have been observed in B-cell chronic lymphocytic leukemia (CLL) (122). Some miRNAs can regulate the TNF/TNFR gene superfamily in CLL (122). Therefore, the identification of the cross-talk between TNF-α and miRNAs could show promising effects for chemotherapeutic agents to control the TME.

## Clinical Uses of TNF-α and Its Importance in Drug Delivery

Carswell et al. discovered in 1975 TNF in the serum of bacilli Calmette-Guérin infected mice inoculated with endotoxin and found that “the substance” induced *in vivo* hemorrhagic necrosis in sarcoma. The substance was named “tumor necrosis factor” (123). Since then, the clinical utility of TNF-α is limited due to its severe systemic toxicity. To mitigate systemic toxicities, the application of TNF-α in cancer treatments became significantly more sophisticated. Its combined use with chemotherapeutic agents in isolated limb perfusion (ILP) has shown good results in tumor response and limb salvage in patients with soft tissue sarcomas (15) and regression in locoregional metastatic melanoma of a limb (14). Because ILP uses arterial and venous canulation with a closed extracorporeal circuit, high doses of TNF- α and chemotherapy can be used in the limb with minimal systemic toxicity. However, the ILP system is cumbersome and does not address its limitation to use TNF-α systemically to improve the efficacy of cancer treatments. The work by our group has shown that when attached to gold nanoparticles (CYT-6091), recombinant human TNF-α (rhTNF) can be given systemically without dose-limiting toxicities up to 3 times that of intravenously-administered unbound rhTNF in phase I clinical trial (124). Although we demonstrated the safety of gold nanomedicine carrying TNF-α in humans, the anti-cancer efficacy was limited, with only a partial response in 27 evaluable participants with advanced solid cancers (124). To prove the concept that this cytotoxic agent was needed to improve the treatment efficacy, paclitaxel analog was added to the gold nanomedicine carrying rhTNF (CYT-21625) and the treatment efficacy, drug delivery efficiency, and systemic toxicities were assessed in multiple mouse models with pancreatic neuroendocrine tumors (pNET) and metastatic aggressive thyroid cancers. We demonstrated significantly improved treatment efficacy across all mouse models treated with CYT-21625 compared to mice treated with intravenous paclitaxel or CYT-6091, with no detectable systemic toxicities or histologic evidence of normal tissue damage or vascular leakage (125). The principle behind its efficacy is related to its effect on tumor vasculature and overcoming high intratumoral interstitial fluid pressure (IFP), allowing for more efficient drug delivery and resulting in markedly increased intratumoral concentrations of paclitaxel (126).

TNF-α has a dose-dependent effect on vascular endothelial cells, inducing angiogenesis at low levels and inhibiting or disrupting it at high levels (127). Neoplasms, just as do healthy tissues, require a blood supply to provide nutrients and allow for growth. In contrast with normal healthy vasculature, neovascular growth in cancers is imperfect, exhibiting a non-continuous endothelium and a sporadically present or absent basement membrane, which increases vessel permeability (16). This increased permeability, along with high cell density, poor venous and lymphatic outflow, and an abnormal ECM contribute to high intratumoral hydrostatic and osmotic IFP (128, 129). Elevated IFP is a barrier to efficient drug delivery in solid tumors as drug cannot effectively travel against a pressure gradient to achieve local therapeutic levels. TNF-α mitigates this barrier by disrupting tumor vasculature, reducing tumor IFP, and allowing chemotherapeutic agents to diffuse into the TME. It disrupts the vasculature by inducing endothelial cell apoptosis, specifically in the tumor while having minimal effect on native healthy vessels due to the differential expression of TNF-R1 in the cancer neovascular network. Increased levels of TNF-R1 in neovascular endothelium has been shown in several tumor types, including thyroid cancer and pNET (125).

Vascular disruption leading to the reduction in intratumoral IFP is the key concept behind the therapeutic uses of TNF-α to improve drug delivery efficiency in cancer treatments. Its most common clinical application is in ILP for locoregionally metastatic melanoma or soft tissue sarcomas of the limb. In this procedure, the limb is isolated with a tourniquet and perfused with oxygenated blood from an extracorporeal circuit, similar in concept to extracorporeal membrane oxygenation for cardiopulmonary failure. Within the perfusion circuit, hyperthermic high-dose chemotherapy (typically the alkylating agent melphalan) is infused with TNF-α. TNF-α serves two functions – inducing hemorrhagic necrosis of the tumor by disrupting the vasculature and allowing the melphalan to locally accumulate in higher levels that would otherwise cause severe systemic toxicities. The limb isolation minimizes the systemic toxicity of both the chemotherapeutic agent and TNF-α –as TNF-α is an acute phase reactant and inflammatory cytokine and can lead to distributive shock – and allows for a several-fold increase in concentration. ILP with melphalan and TNF-α was initially shown to be effective in a phase II trial, which resulted in 21 of 23 complete responses and two partial responses with no patient experiencing treatment failure (130). This cumbersome treatment strategy is only feasible in limb lesions and is not an option for primary or metastatic cancers in solid organs.

Recent work by our group has employed the concept of TNF-α as a facilitator of systemic drug delivery with a novel use of gold nanomedicine. Gold nanoparticles passively target solid tumors by the enhanced permeability and retention effect, preferentially accumulating in their tissue through a combination of highly permeable vasculature and the larger size of the particles compared to molecules dissolved in plasma (131, 132). Gold nanomedicine has a harder time passing through the tight junctions of normal vascular endothelium in non-neoplastic tissue and selectively extravasates into the TME, where it accumulates (132). In addition, the gold nanoparticles carrying rhTNF actively targets cancer neovasculature by binding to the differentially expressed TNF receptors on tumor neovascular endothelium (125). As is the case for ILP, this modality of TNF-α delivery reduces its toxic systemic effects through both active and passive tumor-specific targeting and allows for higher concentrations to be delivered. To improve treatment efficacy in cancers, we demonstrated a significantly lower tumor burden across multiple *in vivo* models using combined rhTNF and paclitaxel analog bound nanomedicine over both rhTNF nanomedicine alone and IV paclitaxel alone in anaplastic thyroid cancer and pNET with no apparent systemic toxicity, further indicating increased efficacy (125). In addition, radiographic imaging studies and histology showed the gold nanomedicine carrying rhTNF only and rhTNF with paclitaxel analog preferentially and specifically targeted tumor tissue and induced vascular leakage only in tumor tissue (125). The transgenic mouse model with pNET showed selective extravasation of MRI contrast in the pancreatic area, corresponding with 18F-FDG-avid lesions, from mice treated with rhTNF bound nanoparticles but not in mice treated with paclitaxel alone and vehicle control. We observed no evidence of extravasation in normal tissue, indicating that the TNF-α induced tumor-specific vascular damage (125). To demonstrate the applicability in a broader range of cancer, a pilot study showed 100% survival *in vivo* in mice with pancreatic ductal adenocarcinoma treated with gold nanomedicine carrying rhTNF-followed by intravenous (IV) paclitaxel compared to 50% survival in the IV paclitaxel only group and 0% in the control group at 42 days (126).

## Conclusion

TNF-α plays a critical role in tumor signaling pathways and immune cell manipulation within the TME. Since Carswell discovered the cytokine in 1975, our understanding of its role in cancers and chronic inflammatory diseases has improved, resulting in the development of treatments that specifically target systemic and TME-related immune cellular response. However, the clinical application such as TNF receptor blockade is only limited to the treatment of chronic inflammatory diseases. Although preclinical data of TNF-α treatment in cancers to improve drug delivery is promising, the treatment efficacy in cancers is not known due to the lack of phase II clinical trials. Because TNF-α induces diverse effects in TME, both oncogenic and tumor-suppressive effects, further studies are warranted to fully understand and selectively induce the anti-tumor effect to improve treatment efficacy in patients with TNF-α sensitive cancers.

## Author Contributions

All authors contributed to the article and approved the submitted version.

## Funding

The preparation of this manuscript was supported by the NIH intramural research grant (# ZIA BC 011286).

## Conflict of Interest

The authors declare that the research was conducted in the absence of any commercial or financial relationships that could be constructed as a potential conflict of interest.

The handling editor declared a shared affiliation with the authors at the time of the review.
